# MicroRNA-204 increases sensitivity of neuroblastoma cells to cisplatin and is associated with a favourable clinical outcome

**DOI:** 10.1038/bjc.2012.425

**Published:** 2012-09-25

**Authors:** J Ryan, A Tivnan, J Fay, K Bryan, M Meehan, L Creevey, J Lynch, I M Bray, A O'Meara, L Tracey, A M Davidoff, R L Stallings

**Affiliations:** 1 Department of Molecular and Cellular Therapeutics, Cancer Genetics Research Group, Royal College of Surgeons in Ireland (RCSI), York House, York Street, Dublin 2, Ireland; 2 National Children’s Research Centre (NCRC), Our Lady’s Children’s Hospital, Crumlin, Dublin 12, Ireland; 3 Department of Surgery, St. Jude Children’s Research Hospital, 262 Danny Thomas Place, Memphis, TN 38105-3678, USA

**Correction to**: *British Journal of Cancer* (2012) **107**, 967–976; doi:10.1038/bjc.2012.356

Upon publication online and in print, the authors realised that a contributor to the original data for this manuscript had been omitted from the authorship of the final paper.

The authors and publishers are now happy to present the complete, full authorship above.

